# Complete Genome Sequences of the Soil Oxalotrophic Bacterium Cupriavidus oxalaticus Strain Ox1 and Its Derived mCherry-Tagged Strain

**DOI:** 10.1128/mra.00181-22

**Published:** 2022-08-04

**Authors:** Fabio Palmieri, Pauline Udriet, Shannon L. Johnson, Karen Davenport, Patrick S. G. Chain, Saskia Bindschedler, Pilar Junier

**Affiliations:** a Laboratory of Microbiology, Institute of Biology, University of Neuchâtel, Neuchâtel, Switzerland; b Bioscience Division, Los Alamos National Laboratory, Los Alamos, New Mexico, USA; University of Southern California

## Abstract

Here, we report the complete genome sequences of the soil oxalotrophic bacterium Cupriavidus oxalaticus Ox1 and a derived mCherry-tagged strain. The genome size is approximately 6.69 Mb, with a GC content of 66.9%. The genome sequence of *C. oxalaticus* Ox1 contains a complete operon for the degradation and assimilation of oxalate.

## ANNOUNCEMENT

Oxalotrophy is the ability to use oxalate as a carbon and energy source. So far, this metabolism has been described in a specialized group of both aerobic and anaerobic bacteria (1, 2). Oxalic acid transformation involves the decarboxylation of oxalate into formate by the formyl-coenzyme A (formyl-CoA) transferase (Frc; EC 2.8.3.16) and the oxalyl-CoA decarboxylase (Oxc; EC 4.1.1.8) (1, 3), followed by formate oxidation by the formate dehydrogenase (4). Excretion of formate through the oxalate/formate antiporter OxlT is required for energy production (5). Cupriavidus oxalaticus Ox1, formerly Pseudomonas oxalaticus, is a soil bacterium isolated from the gastrointestinal tract of Indian earthworms (6). This species has been used as a model to study oxalotrophy through enzymatic studies (7–10). In contrast, analysis of the genes encoding the key enzymes involved in oxalotrophy is still lacking. Here, we sequenced and annotated the complete genome of *C. oxalaticus* strains Ox1 NEU 1047 (wild type) and NEU 1287 (mCherry tagged), to study oxalotrophy on the former and to check the chromosomic insertion of the mCherry fluorescent protein tag for the latter. We report the presence of a putative complete oxalotrophy transcriptional operon. In addition, we confirmed the chromosomic insertion of *mCherry* upstream of the *glmSU* genes (11, 12) in the constitutive fluorescently tagged mutant (strain NEU 1287 mCherry), which was prepared in-house using a mini-Tn7-mCherry transposon system (13).

The strains were cultured in nutrient broth at 37°C under constant agitation (120 rpm) overnight. Genomic DNA was extracted using the Genomic-tip 20/G kit (Qiagen GmbH, Germany), following the manufacturer’s instructions. Both bacterial genomes were sequenced and assembled by the Lausanne Genomic Technologies Facility (University of Lausanne). Genomic DNA was sheared using a Megaruptor instrument (Diagenode, Denville, NJ, USA) to obtain 10- to 15-kb fragments. After shearing, the DNA size distribution was checked on a fragment analyzer (Advanced Analytical Technologies, Ames, IA, USA). DNA (500 ng) was used to prepare several SMRTbell libraries with the PacBio SMRTbell Express template prep kit v2.0 (Pacific Biosciences, Menlo Park, CA, USA) according to the manufacturer’s recommendations. DNA fragments of <3 kb were size selected using AMPure PacBio beads. The DNA was sequenced using v3.0/v3.0 chemistry and diffusion loading on a PacBio Sequel I instrument with a movie length of 600 min and a preextension time of 120 min using one single-molecule real-time (SMRT) cell 1M v3. *De novo* microbial assembly was performed using SMRT Link v9.0 with the Microbial Assembly Workflow v1.0.4, which includes a preassembly automatic quality-filtering step. Default parameters were used, except for the genome length, which was set to 6 Mb instead of 5 Mb. The workflow reported rotation to the *oriC* position and circular status of the contig. The genomes were not determined to be complete manually. Genome annotation was carried out with using the NCBI Prokaryotic Genome Annotation Pipeline (PGAP) (14). Assembly and annotation statistics for both strains are provided in Table 1.

**TABLE 1:** Assembly and annotation statistics for *C. oxalaticus* Ox1 and *C. oxalaticus* Ox1 mCherry

Characteristic	*C. oxalaticus* Ox1 (NEU 1047)	*C. oxalaticus* Ox1 mCherry (NEU 1287)
Genome size (bp)	6,694,750	6,697,997
No. of chromosomes	2	2
No. of contigs	2	2
*N*_50_ (bp)	3,885,446	3,888,701
Mean coverage (×)	189.27	160.97
GC content (%)	66.94	66.94
Total no. of genes	6,059	6,064
Total CDSs^*a*^	5,975	5,980
No. of protein-coding CDSs	5,872	5,872
No. of rRNAs (5S, 16S, 23S)	5, 5, 5	5, 5, 5
No. of tRNAs	65	65
GenBank accession no.	CP069811.1, CP069812.1	CP069809.1, CP069810.1
GenBank assembly accession no.	GCA_016894385.1	GCA_016894365.1

a CDSs, coding DNA sequences.

### Data availability.

This whole-genome sequencing project has been deposited at GenBank under the BioProject accession no. PRJNA695296. The nucleotide sequences and genome assembly accession numbers for both *C. oxalaticus* Ox1 and Ox1 mCherry are presented in Table 1.
